# Influence of the toxicological status on the diagnosis of fatal drowning

**DOI:** 10.1007/s00414-023-03057-3

**Published:** 2023-07-11

**Authors:** Clément Poulain, Olivier Mathieu, Valérie Gouetta, Éric Baccino, Pierre-Antoine Peyron

**Affiliations:** 1 Department of Forensic Medicine, University Hospital of Montpellier, Montpellier, France; 2 Department of Medical Pharmacology and Toxicology, University Hospital of Montpellier, Montpellier, France; 3 SNPS- Laboratoire de Police Scientifique de Toulouse, Toulouse, France; 4 Montpellier Criminal Law and Forensic Sciences research team (EDPFM, UR-UM212), Univ. Montpellier, Montpellier, France

**Keywords:** Drowning, Toxicology, Diatom test, Xenobiotics, Autopsy, Forensic medicine

## Abstract

Drowning is the leading cause of death by accident of everyday life in people under 25 years of age. Xenobiotics are frequently involved in drowning cases but their influence on the diagnosis of fatal drowning has not been studied so far. This preliminary study aimed to assess the influence of an alcohol and/or a drug intoxication on the autopsy signs of drowning, and on the results of diatom analyses in drowning deaths. Twenty-eight autopsy cases of drowning including 19 freshwater drownings, 6 seawater drownings, and 3 brackish water drownings were prospectively included. Toxicological and diatom tests were performed in each case. The influence of alcohol and other xenobiotics on drowning signs and diatom analyses was assessed separately then in combination through a global toxicological participation score (GTPS). Diatom analyses showed positive results in lung tissue in every case. No significant association was found between the degree of intoxication and the diatom concentration in the organs, even after considering freshwater drowning cases only. The vast majority of the traditional autopsy signs of drowning were not significantly affected by the individual toxicological status either, with the exception of lung weight which tended to raise in case of intoxication, probably due to the pulmonary edema and congestion increase. Further research on larger autopsy samples is needed to confirm the results of this exploratory study.

## Introduction

Drowning is defined as the process of experiencing respiratory impairment from submersion/immersion in liquid [1]. According to a recent national survey conducted by Santé Publique France [2], it is the leading cause of accidental death in people under 25 years of age in France, which makes it a major public health issue. During the 2021 summer period, 44% of fatal accidental drownings took place in the sea, 15% in swimming pools, 39% in rivers or lakes and 2% in other places (bathtubs, ponds, etc.). Moreover, 85% of the 1753 drownings studied were accidental, 10% intentional (suicide, assault) and 5% of unknown origin.

Deaths by drowning are by definition violent deaths and should therefore systematically be investigated by a judicial inquiry including a forensic autopsy, according to current European recommendations [3]. In addition to searching for signs that could suggest the physical intervention of a third party, or a previous medical condition that could have contributed to the drowning, the main mission of the forensic pathologist consists in confirming the drowning and differentiating it from a submersion without active water inhalation. The latter condition can result from several circumstances such as a sudden death before or after immersion (e.g., due to a cardiac pathology), a fatal trauma in close proximity to an aquatic environment, or attempts to conceal a lifeless body in the context of a homicide. For this purpose, several signs are looked for at the autopsy such as pulmonary edema, frothy sputum, visceral congestion, liquid effusions in the serous membranes, or water in the stomach [4–6]. However, none of these signs is pathognomonic, and their absence does not eliminate the diagnosis of drowning.

Because of the poor diagnostic performance of the autopsy signs of drowning, several ancillary methods have been proposed to confirm the diagnosis, among which the diatom analysis in tissues [7, 8], which currently represents the gold standard [9]. Diatoms are ubiquitous siliceous algae theoretically found in any wet environment. Their small size (2 μm to 1 mm) allows them to cross the alveolar-capillary barrier and to be disseminated in the organs by the blood flow. The presence of diatoms in pulmonary tissue is considered very sensitive, but not very specific because of a possible post-mortem penetration of water in the tracheobronchial lumen. Conversely, their presence in solid organs is considered to be not very sensitive but specific, as diatoms could only have naturally penetrated the organs via the blood flow generated by a beating heart. Many authors agree that the diagnosis of drowning can be given if concordant diatom taxa are found in solid organs and in water. However, several studies have shown significant discrepancies in the validity and reliability of diatom analysis, therefore the scientific community remains divided regarding the diagnostic performance of this method [7, 9–15].

Given the absence of reliable and consensual markers of fatal drowning, this diagnosis still remains a diagnosis of exclusion usually made after researching intrinsic (pathological) or extrinsic (traumatic, toxicological) factors that may have played a role in the mechanism of death [4–6]. Some of these factors may be responsible for so-called “atypical” drownings, which are characterized by a rapid onset of death and therefore by particularly poor signs at autopsy. Among these factors, the toxic status of the victim at the time of drowning is an important one to be taken into consideration. In the 2021 French survey, evidence of an alcohol consumption, or of a suspected consumption, was found in 8% of all drownings, and proportion of fatal drownings was greater in cases of alcohol consumption (37% vs. 21% without alcohol) [2].

Other xenobiotics are frequently associated with drowning in forensic practice, such as recreational drugs in the context of accidental drowning, or drugs for suicidal or therapeutic purposes. These xenobiotics can cause various deleterious effects on the organism such as central nervous system depression, respiratory depression or modulation of the cardiac activity. They are also known to influence the mechanism of drowning (for example, asphyxia-related forms of drowning have been empirically more frequently described in alcohol-impaired individuals).

Their action is thus likely to participate in the determinism of death, and may potentially compromise the diagnosis of drowning deaths by affecting the occurrence of autopsy findings and the distribution of diatoms in the body.

The aim of this preliminary study was therefore to assess the influence of an alcohol and/or a drug intoxication on the autopsy signs of drowning, and on the results of diatom tests in drowning deaths.

## Materials and methods

### Study design and population

This exploratory study prospectively included bodies found immersed in water or near an aquatic environment and suspected of being drowned, subsequently autopsied at the Forensic Institute of Montpellier, France, between June 2020 and March 2022. Bodies whose cadaveric decay did not allow tissue sampling of interest were not included.

Diagnosis of drowning was made after the elimination of other potential causes of death, based on all available data gathered before (investigation data), during and after (histological and toxicological findings, results of strontium analyses) the autopsy.

### Collected data

At autopsy, the following data were systematically collected: sex, age, anthropometric data (weight, height), type of drowning water (sea, fresh or brackish water), autopsy signs of drowning (foam, lung weight, pulmonary edema, lung inflation, pleural effusion, pericardial effusion, peritoneal effusion, water in the digestive tract).

### Sampling procedure

For toxicological analyses, samples of cardiac blood, femoral blood, vitreous humor, and urine were taken. For diatom analyses, 10g-fragments of lung were collected in situ using single-use sterile clamps and scalpels. All samples were kept at -30°C until analysis.

### Toxicological analyses

The reference toxicological analyses were systematically carried out, as prescribed in the article A.43-6-2 of the French code of criminal procedure [16]. The main xenobiotics (ethanol, narcotics [cannabinoids, amphetamines, cocaine, opiates and metabolites], psychoactive drugs [hypnotics, anxiolytics, neuroleptics and antidepressants], non-psychoactive drugs [beta-blockers, antiarrhythmics, anesthetics, anticoagulants, antidiabetics, analgesics, antiparkinsonians], and other xenobiotics) were searched and quantified by spectrometry and chromatography (HS-GC-FID, LC-MS/MS, LC-20AD).

Blood alcohol concentration (BAC) was considered to be positive for concentration > 0.3 g/l, or > 0.5 g/l in case of presence of volatile substances related to putrefaction, to minimize the risk of falsely elevated BAC due to a putrefactive process [17]. Alcohol concentration was considered significantly high if > 2.0 g/l. Each subject was thus classified into one of the three following groups:

- Negative BAC.
- Positive BAC.
- Very high BAC.

Quantitative thresholds were defined for each positive xenobiotic (other than alcohol) according to the literature [18–21], allowing to classify the subjects into three other groups:

- Negative toxicology.
- Infra-therapeutic or therapeutic concentrations.
- Supra-therapeutic or toxic concentrations.

When only resuscitation drugs were detected, subjects were included into the “negative toxicology” group.

A global toxicological participation score (GTPS) was established, combining the alcohol and the xenobiotic classifications (Alcohol = 0 if negative 1 if positive, 2 if very high; xenobiotics = 0 if negative, 1 in case of therapeutic or infra-therapeutic concentrations, 2 in case of supra-therapeutic or toxic concentrations). A GTPS = 1 was considered as a score suggesting a possible toxicological contribution to death, and a GTPS ≥ 2 as a probable toxicological contribution to death.

### Diatom analyses

Because of its better morphological analysis of diatoms compared to photonic microscopy, diatom tests were performed on lung tissue by scanning electron microscopy reading (complete reading of the disk until reaching 1000 diatoms) after acid extraction, allowing to classify the subjects in three groups according to the concentration of extracted diatoms (positive analysis [> 150/10g], low positivity [20-150/10g], and negative analysis [<20/10g])

### Statistical analysis

Qualitative variables were expressed as numbers with their associated percentages. Groups were compared using Fisher’s test. Quantitative data were tested for normality using the Kolmogorov-Smirnov test, and expressed as mean and standard deviation, or median and interquartile range. Groups were compared using the unpaired *t*-test and ANOVA for variables with a normal distribution, and the Wilcoxon Mann-Whitney test or Kruskall-Wallis test for the other variables. Right and left lung weights were added together to allow for a single analysis per case. As the sample size was small, we also performed analyses by merging the negative and low positive groups; as well as the low positive and strongly positive groups of each classification. The significance level was set for a *p*-value at 5%, with a tendency to significance between 5 and 10%. Statistical analyses were performed using GMRC shiny stats.

## Results

Of the 35 cases initially meeting inclusion criteria, 6 cases had to be excluded: 3 for which toxicology had not been performed, and 3 because of diatom contamination. Of the 29 remaining cases, 1 case was diagnosed as a non-vital drowning and was therefore excluded (hypertrophic cardiomyopathy resulting in a probable rhythm disorder, in a diver who fainted in front of a witness, while getting into water, without being submerged)﻿﻿﻿﻿﻿﻿﻿﻿ ﻿(﻿﻿F﻿﻿i﻿g. 1). The characteristics of the twenty-eight cases finally included are described in Table 1.

**Fig. 1:**
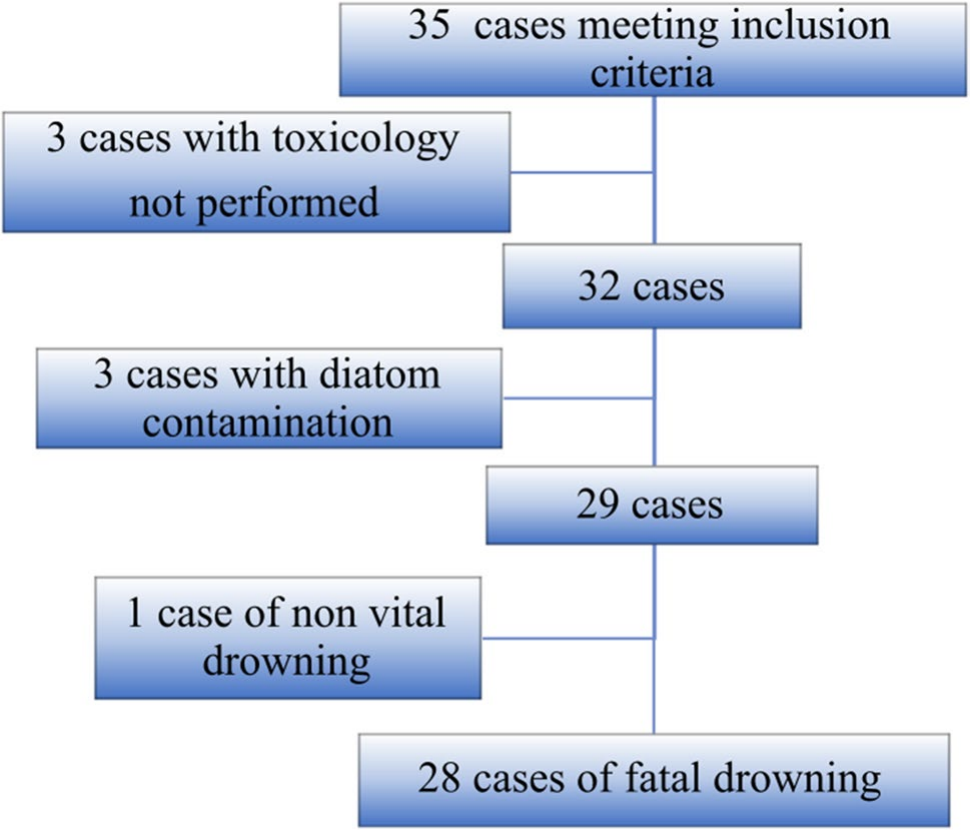
Flow chart of the case selection process

**Table 1:** Drowning case characteristics (*n*=28) and environment

Age in years (mean ± standard deviation)	39.8 ±21.9
0-19y	5 (16.6%)
20-64y	17 (60.7%)
≥65y	6 (21.4%)
Sex	
Male	19 (67.9%)
Female	9 (32.1%)
Sex ratio	2.1
Weight in kg (median [Q25;75])	69.0 [62.2; 79.5]
Height in cm (median [Q25;75])	168.0 [158.0; 174.0]
Drowning water	
Freshwater	19 (67.8%)
Seawater	6 (21.4%)
Brackish water	3 (10.7%)

The most frequent place of drowning was freshwater (*n*=19; 67,8%). The manner of death was mostly accidental (53,6%), then undetermined (39,3%) and lastly suicidal (7,1%). Ten cases (35,7%) showed signs of cadaveric alteration at the autopsy, mostly at a moderate stage (greenish coloration of the skin). A case of complex suicide displayed thoracic and laryngeal injuries that allowed a sufficient survival time for subsequent drowning. Only 3 cases (10,7%) had a known history of cardiovascular disease (atherosclerotic disease and aortic stenosis). No other relevant condition (epilepsy, pulmonary disease) was known among the included subjects.

## Autopsy findings (Table 2)

Foam in the airways was observed in half of drowning cases (50,0%), but only in 32,1% as a complete formation of external foam. Pulmonary edema and pleural effusion were the most frequent signs (85,7%). Lung inflation was described in 19 cases (67,9%), always associated with pulmonary edema (emphysema aquosum). Water was present in the digestive tract (stomach and/or bowel) in 8 cases (28,6%).

**Table 2:** Autopsy findings (*n*=28)

Foam	
External foam	9 (32.1%)
Foam in the airways	14 (50%)
Lung weight (g) (median [Q25;75])	959 [798 ; 1325]
Pulmonary edema	24 (85.7%)
Inflated lungs	19 (67.9%)
Pleural effusion	24 (85.7%)
Pericardial effusion	10 (35.7%)
Peritoneal effusion	4 (14.3%)
Water in the digestive tract (stomach and/or bowel)	8 (28.6%)

### Toxicological analyses (Fig. 2)

Alcohol was found in 11 cases (39,3%), with 3 cases (10,7%) considered significantly intoxicated (> 2.0 g/l). Xenobiotics other than alcohol were found in 8 cases (28,5%), including 2 cases (7,1%) with supra-therapeutic or toxic concentrations. Toxicological analyses found benzodiazepines (*n*=4), antidepressants (*n*=2), bisoprolol (*n*=1), pipamperone (*n*=1), carbamazepine (*n*=1), morphine (*n*=1), and tetrahydrocannabinol (*n*=3). The GTPS revealed a possible toxicological participation in 10 cases (35,7%), and a probable toxicological participation in 6 cases (21,4%).

**Fig. 2:**
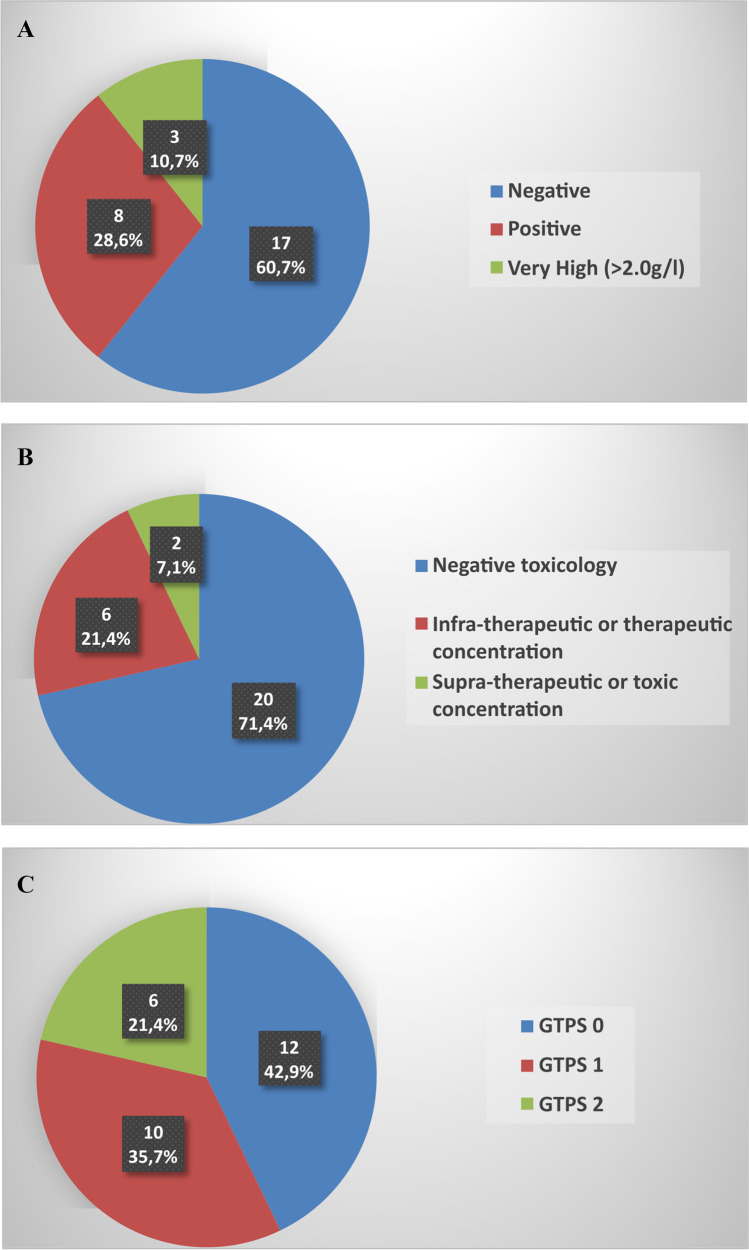
Toxicological analyses classifications: alcohol (OH) analyses (**a**), xenobiotics (other than alcohol) analyses (**b**), and Global Toxicological Participation Score (**c**)

### Diatom analyses

Nine cases (32,1%) were positive for diatoms, and 19 cases (67,9%) had low concentrations of diatoms. Three of the positive cases (10,7%) were children *(<15 year*s), each of them being negative for toxicology, and having low positivity criteria for diatoms. There were no negative diatom results.

### Influence of the toxic status

The different toxic status groups described below did not differ significantly regarding age, sex, weight, height and immersion environment (*p*>0.05).

### Diatoms

The influence of the toxic status on diatom tests is summarized in Table 3. There were no statistically significant differences between groups, even after pairing groups or considering freshwater drowning cases only.

**Table 3:** Diatom analyses results according to the toxicological status (*n*=28)

		Diatoms		
		Positive (*n*=9)	Low positive (*n*=19)	
Alcohol	Negative	4 (23.5%)	13 (76.4%)	*p*=0.24
	Positive	3 (37.5%)	5 (62.5%)	
	Very high	2 (66.7%)	1 (33.3%)	
Xenobiotics	Negative	6 (30%)	14 (70%)	*p*=0.18
	Infra- therapeutic or therapeutic	1 (16.7%)	5 (83.3%)	
	Supra- therapeutic or toxic	2 (100%)	0	
GTPS	GTPS 0	3 (25%)	9 (75%)	*p*=0.17
	GTPS 1	2 (20%)	8 (80%)	
	GTPS ≥ 2	4 (66.7%)	2 (33.3%)	

*GTPS* global toxicological participation score

### Autopsy signs—alcohol (Table 4)

Presence of foam (either external or in the airways), pulmonary edema, lung inflation, pleural effusion, pericardial effusion, peritoneal effusion and water in the digestive tract did not show significant differences between groups. Lung weight was found to be higher with the presence of alcohol (*p*=0.047) after pairing the positive and the highly positive groups.

**Table 4:** Drowning signs according to blood alcohol concentrations (BAC) (*n*=28)

	Negative *n* =17	Positive *n* =8	Very high *n*=3	*p*
Foam				
External Foam	6 (35.3%)	2 (25%)	1 (33.3%)	1.000
Foam in the airways	8 (47.1%)	5 (62.5%)	1 (33.3%)	0.74
Lung weight (g) (median [Q25;75])	940 [745 ; 1006]	1462 [806; 1713]	1300 [1121; 1350]	0.14
Pulmonary edema	15 (88.2%)	6 (75%)	3 (100%)	0.73
Inflated lungs	12 (70.6%)	5 (62.5%)	2 (66.7%)	1.000
Pleural effusion	15 (88.2%)	6 (75%)	3 (100%)	0.73
Pericardial effusion	6 (35.3%)	2 (25%)	2 (66.7%)	0.35
Peritoneal effusion	4 (23.5%)	0	0	0.39
Water in the digestive tract (stomach and/or bowel)	5 (29.4%)	2 (25%)	1 (33.3%)	1.000

### Autopsy signs—xenobiotics except alcohol (Table 5)

There were no significant differences between groups concerning the majority of autopsy signs, except for pleural effusion (*p*=0.01) and water in the digestive tract (*p*=0.034). However, these differences were no longer observed when comparing the negative group with both positive groups (*p*>0.05). Lung weight tended to be increased in case of xenobiotic consumption when using this classification (*p*=0.09).

**Table 5:** Drowning signs according to xenobiotic concentrations (*n*=28)

	Negative *n* =20	Therapeutic or infra-therapeutic *n* =6	Supra-therapeutic or toxic *n*=2	*p*
Foam				
External Foam	6 (30%)	2 (33.3%)	1 (50%)	1.000
Foam in the airways	11 (55%)	2 (33.3%)	1 (50%)	0.82
Lung weight (g) (median [Q25;75])	893 [731; 1266]	1160 [977; 1578]	1360 [1150; 1570]	0.24
Pulmonary edema	17 (85%)	5 (83.3%)	2 (100%)	1.000
Inflated lungs	14 (70%)	3 (50%)	2 (100%)	0.53
Pleural effusion	19 (95%)	5 (83.3%)	0	<0.01*
Pericardial effusion	7 (35%)	3 (50%)	0	0.68
Peritoneal effusion	4 (20%)	0	0	0.67
Water in the digestive tract (stomach and/or bowel)	6 (30%)	0	2 (100%)	0.034*

* p<0.05

### Autopsy signs—GTPS (Table 6)

There were no significant differences between groups for the majority of autopsy signs, except for lung weight (*p*=0.03). Pleural and peritoneal effusions tended to be less observed in case of positive GTPS (*p*=0.09 and *p*=0.056, respectively). Lung weight was still significantly increased (*p*<0.01) and peritoneal effusion less observed (*p*=0.024) after fusing GTPS 1 and 2, in comparison with GTPS 0.

**Table 6:** Drowning signs according to the global toxicological participation score (GTPS) (*n*=28)

	GTPS 0 *n* =12	GTPS 1 *n* =10	GTPS 2 *n* = 6	*p*
Foam				
External Foam	4 (33.3%)	2 (20%)	3 (50%)	0.44
Foam in the airways	7 (58.3%)	4 (40%)	3 (50%)	0.88
Lung weight (g) (median [Q25;75])	816 [646 ; 971]	1160 [803 ; 1482]	1350 [1032 ; 1618]	0.03*
Pulmonary edema	11 (91.7%)	7 (70%)	6 (100%)	0.31
Inflated lungs	10 (83.3%)	4 (40%)	5 (89.3%)	0.078
Pleural effusion	12 (100%)	8 (80%)	4 (66.7%)	0.09
Pericardial effusion	4 (33.3%)	4 (40%)	2 (33%)	1.000
Peritoneal effusion	4 (33.3%)	0	0	0.056
Water in the digestive tract (stomach and/or bowel)	4 (33.3%)	1 (10%)	3 (50%)	0.21

* p<0.05

Subgroup analyses of freshwater drowning cases only (*n*=19) did not show significant differences for any parameter.

## Discussion

This preliminary study aimed at assessing the impact of an alcohol and/or a drug intoxication on the autopsy findings and on the results of diatom analyses in cases of vital drowning. In cases of a high degree of xenobiotic exposure, intoxication may represent a competing cause of death or a major stress factor that might be expected to shorten the fatal period of drowning (i.e., terminal/secondary drowning mediated by poisoning). To the best of our knowledge, the influence of such a factor has not been investigated so far in forensic research. This study was based on 28 autopsy cases for which toxicological and diatom tests were systematically performed. Our population was mainly composed of middle-aged men, which is in line with the characteristics of the drowned population reported in France in 2021 [2].

Although our study was carried out in a Mediterranean coastal region, sea water drownings accounted for only 21,4% of the cases included whereas there were most prevalent in the 2021 French epidemiological survey (44%). These discrepancies may be explained by the fact that this survey was conducted during the summer period. Bathing practices are therefore different from those of our study which was not restricted to a specific season.

The prevalence of drowning signs in our study was consistent with the literature data [22]. In particular, external foam was noted in 32,1% of cases (13.5 to 40% in literature), and pulmonary edema was associated with lung inflation *(*emphysema aquosum*)* in 67.9% of cases (30 to 97% in literature).

Compared to the data from the 2021 epidemiological survey, which included fatal and non-fatal drownings, the proportion of alcohol consumption was higher in our population (39% vs. 8%), which is consistent with the higher proportion of deaths reported in cases of drowning after alcohol consumption [23, 24]. Drugs were also more prevalent in our study (10,7% vs. less than 1%). These differences can also be explained by the fact that the epidemiological survey was mainly based on declarative data while we performed systematic toxicological analyses.

We first assessed the influence of alcohol and of other xenobiotics on drowning signs and diatom tests separately, then in combination through a global score (GTPS) specifically created for the purpose of this study. Diatom analyses showed positive results in every case, although the majority of cases displayed low positivity criteria (67,9%). No association was found between the results of diatom tests in lung tissue and the presence of alcohol or other drugs, even using GTPS and trying different group combinations or sub-group analyses (on freshwater drowning cases in particular).

The vast majority of autopsy signs showed no significant differences between groups, except for lung weight that was significantly increased when GTPS was increased (*p*=0.03) and in case of alcohol intoxication after pairing both positive groups (*p*=0.047). Moreover, lung weight tended to be higher in case of consumption of xenobiotics (*p*=0.09). Our hypothesis is that the lung edema and congestion generally seen in cases of intoxication [25] are probably combined with those seen in cases of drowning. Both lesional and hemodynamic pulmonary edemas are likely to co-exist in this context.

Pleural and peritoneal effusions have shown some tendencies to be less present in case of toxic impregnation, except in the alcohol group. As the explicative mechanism remains unclear, these findings should be further explored through a larger study. Some other autopsy features of drowning that were not considered in this study could also be further explored and correlated with toxicological findings, such as Sveshnikov’s sign, Ueno's sign, Paltauf’s spots, and aortic hemolytic staining. Given the small number of cases included in this study, the list of autopsy signs to be collected had to be restricted to prevent an inflation of the alpha risk.

A case specifically caught our attention as regards the relevance of diatom test in case of penetrating wounds, particularly in the thoracic region. Such wounds are generally assumed to result in a contamination of the internal organs, thus in biased diatom analyses. In this case of a complex suicide including self-inflicted stab wounds in the left lung before submersion, diatom analyses showed much lower concentrations in the left stabbed lung than in the right lung. We speculate that this result may be explained by a left hypoventilation due to pneumothorax.

Interestingly, on the three cases of swimming pool drownings included in the fresh water drowning group, each showed positive diatom results. These findings refute the belief that urban water systems are devoid of diatoms, and that these latter can sometimes be used as markers of drowning in these environments.

We are aware that this preliminary study presents several limitations. The small sample size probably limited our findings, and possibly did not allow to highlight a significant association between diatom concentrations in lungs and the toxicological status of the subjects. However, this is an exploratory study whose results would need to be confirmed by a multicenter study including more cases. One may argue that a more exhaustive diatom analysis including other organs may also be valuable for a better exploration of the distribution of diatoms. However, diatom test in solid organs is controversial [9] and the concentrations generally observed appears to be too low to establish any threshold to assess the influence of a potential intoxication. As regards diatom concentrations in lung, the threshold for negative results was based on the literature [4] while the low positivity/positivity threshold was arbitrarily chosen based on the experience of the diatom expert. A threshold defined on the basis of a statistical analysis of a large sample would have been more relevant.

As mentioned in the introduction, the potential influence of intrinsic factors (cardiac disease, epilepsy) on the drowning process has been discussed in the literature and has to be taken in consideration [2, 22], as they can be confounding factors for the diagnosis of fatal drowning. However, an exhaustive medical history was frequently missing in our study. As a result, a pre-existing condition (cardiovascular disease) was known or noted in only 3 cases (10,7%), which is less than the prevalence reported in the literature (14 to 49%) [22].

Since the water at the drowning site was not systematically sampled as recommended, its testing could not be included in the diatom analysis, although the concentration of diatoms in water could have affected the interpretation of the diatom analyses. The therapeutic ranges and toxicity thresholds for the various xenobiotics were based on the literature, but the GTPS was defined in an arbitrary way in order to try to take into account the toxicological impact of both alcohol and drugs. In view of the globally more comparable results between the alcohol and the GTPS groups, alcohol seemed to be of greater importance than other xenobiotics in the calculation of the GTPS. In this respect, positive alcohol thresholds may have been defined differently. Perhaps a more complex score based on a larger sample could allow a better consideration of the global intoxication state.

## Conclusion

This exploratory study did not reveal a clear influence of the individual toxicological status on the diagnosis of fatal drowning. In particular, it showed no association between alcohol and/or drug intoxication and the results of diatom tests. The majority of the traditional autopsy signs of drowning did not appear to be significantly influenced by the toxicological status. Lung weight was the only parameter to show an increase when drowning was associated with some toxic consumption, especially with alcohol intoxication. Studies on larger samples are needed to further investigate the hypothesis that a consumption of alcohol and/or drugs may affect diatom analyses, and to further explore the association observed between the toxicological status and lung weight in fatal drownings. Future research should better take into account medical history as well as the diatom concentration in the drowning environment, as the ratio of diatom concentration in the organs to diatom concentration in water might be a marker of interest [15, 26–28].

## Data Availability

The datasets generated during and/or analysed during the current study are not publicly available in order to guarantee anonymity, as this is a single-center study over a short period of time, but are available from the corresponding author on reasonable request.
